# Identification of prognostic RNA editing profiles for clear cell renal carcinoma

**DOI:** 10.3389/fmed.2024.1390803

**Published:** 2024-07-18

**Authors:** Weihong Chen, Shaobin Li, Dongqin Huang, Yuchao Su, Jing Wang, Zhiru Liang

**Affiliations:** ^1^Department of Anxi County Hospital, Quanzhou, China; ^2^Xilin Gol League Central Hospital, Xilin Hot, China

**Keywords:** clear cell renal cell carcinomas, RNA editing, bioinformatics, ccRCC biomarker, ccRCC risk score

## Abstract

**Objective:**

Clear cell renal cell carcinoma (ccRCC) is the most common type of renal cancer and currently lacks effective biomarkers. This research aims to analyze and identify RNA editing profile associated with ccRCC prognosis through bioinformatics and *in vitro* experiments.

**Methods:**

Transcriptome data and clinical information for ccRCC were retrieved from the TCGA database, and RNA editing files were obtained from the Synapse database. Prognostic models were screened, developed, and assessed using consistency index analysis and independent prognostic analysis, etc. Internal validation models were also constructed for further evaluation. Differential genes were investigated using GO, KEGG, and GSEA enrichment analyses. Furthermore, qPCR was performed to determine gene expression in human renal tubular epithelial cells HK-2 and ccRCC cells A-498, 786-O, and Caki-2.

**Results:**

An RNA editing-based risk score, that effectively distinguishes between high and low-risk populations, has been identified. It includes CHD3| chr17:7815229, MYO19| chr17:34853704, OIP5-AS1| chr15:41590962, MRI1| chr19:13883962, GBP4| chr1:89649327, APOL1| chr22:36662830, FCF1| chr14:75203040 edited sites or genes and could serve as an independent prognostic factor for ccRCC patients. qPCR results showed significant up-regulation of CHD3, MYO19, MRI1, APOL1, and FCF1 in A-498, 786-O, and Caki-2 cells, while the expression of OIP5-AS1 and GBP4 was significantly down-regulated.

**Conclusion:**

RNA editing site-based prognostic models are valuable in differentiating between high and low-risk populations. The seven identified RNA editing sites may be utilized as potential biomarkers for ccRCC.

## 1 Introduction

The regulation of blood pressure, elimination of metabolites from bodily fluids, and maintenance of electrolyte balance are all dependent on the kidney, an essential organ in the human body (1). Malignant transformation of the kidney can impair its normal function and pose a significant risk to people’s lives and health (2). Kidney cancer is the second most prevalent malignant tumor of the urinary system, accounting for approximately 3% of all malignant tumors, with a male-to-female patient ratio of around 2:1 (3).

The most common type of kidney cancer, known as clear cell renal cell carcinomas (ccRCC), accounts for around 70 to 80% of cases, and its morbidity and mortality rates have been increasing annually (4). Despite the popularity of clinical therapies such as radiation, chemotherapy, and surgery, studies have shown that these may be ineffective for individuals with advanced ccRCC (5). Furthermore, recurrence rates can reach 40% even after surgery, and 30% of patients with ccRCC have metastases at the time of clinical diagnosis, leading to a poor prognosis (6). As a result, finding biomarkers that may be utilized for early diagnosis and precise prognosis is crucial and has become a popular area of study in recent years.

RNA editing is a post-transcriptional mechanism that changes the sequence of selected RNA transcripts (7, 8). In mammals, the most common type of RNA editing is adenosine to inosine (A-to-I), a molecular process that changes nucleotide sequences of double-stranded RNAs (dsRNAs) by the deamination of the canonical Adenosine (A) base to the Inosine (I) (9). This molecular mechanism is mediated by the Adenosine Deaminases Acting on dsRNA enzymes (ADAR). Three members of this family are encoded in the mammalian genome: ADAR1 (also known as ADAR), ADAR2 (also known as ADARB1) and ADAR3 (also known as ADARB2) (7, 10). When edited RNAs are processed, the ribosomes and splicing machinery decode Inosines as Guanosines instead of the Adenosines encoded in the genome. Editing is classified as “recoding-type editing” if these A → G mismatches occur in protein-coding sequences and lead to non-synonymous substitutions that generate novel protein variants (11, 12). Editing can also occur in non-coding RNAs or non-coding parts of mRNAs generating new protein isoforms by altering the splicing pattern of the pre-mRNA affecting the cellular fate of an mRNA and/or its probability of being translated, by editing of microRNA (miRNA) binding sites in its 3′ untranslated region (UTR) or by directly editing the related miRNAs themselves (10). A-to-I RNA editing plays a significant role in human cancers, which has been widely studied and discussed. Different cancer types exhibit varying levels of ADAR enzyme expression and RNA editing (13). For instance, brain cancers often display low levels of RNA editing (14), while certain thyroid, head and neck, lung, and breast cancers exhibit excessive or misregulated editing (15). These differences may be closely related to the occurrence, development, and treatment response of cancer. Therefore, further understanding of the role of ADAR enzymes and RNA editing in cancer is crucial for developing new cancer treatment strategies.

One of the hallmarks of normal cells is the association between RNA and proteins, as well as the accurate translation of proteins. RNA is typically stable. Many types of cancer, such as hepatocellular carcinoma, lung carcinoma, and breast carcinoma, have been linked to increased RNA editing during cancer development (16, 17). The abnormal increase in RNA editing disrupts biological balance during cancer development, leading normal cells to become cancerous (18). Imbalances in A-to-I RNA editing catalyzed by ADAR1 are associated with cancer. Through rigorous bioinformatics methods, identified differential RNA editing sites (DES) related to low or high sensitivity, which were validated using breast cancer (BC) cell lines. In BC patients found that DES was primarily present in immune response genes, and a significant association was observed between RNA editing levels in the genes LSR, SMPDL3B, HTRA4, and LL22NC03-80A10.6 and progression-free survival (19). A detailed analysis of the oncogenic mechanisms of A-to-I RNA editing events in 33 cancer types covered in the Cancer Genome Atlas was conducted. For individual candidates among approximately 1,500,000 quantitative RNA editing events, a variety of downstream functional annotations were performed. Identified 24,236 A-to-I RNA editing events with potential functions, involving several key genes and molecules such as APOL1, IGFBP3, GRIA2, BLCAP, and miR-589-3p (20).

After in-depth research, scientists have identified hundreds of A-to-I RNA editing sites, which are specifically labeled as differential editing sites because they are closely associated with clinical outcomes of cancer (21). The study reveals significant differences in the editing of ubiquitination sites between tumor and non-tumor samples, as well as between different tumor subtypes in the TCGA dataset. This difference is not only reflected in the editing frequency, but more importantly, it is closely related to the clinical outcomes of cancer. Non-synonymous editing sites on genes such as GSTM5, WDR1, SSR4, and PSMC4 have become the focus of research in this field. These editing changes at these sites may serve as important biomarkers for predicting cancer progression and treatment effectiveness (22). Through in-depth analysis of small RNA sequencing data from 154 patients with ccRCC and 22 normal control kidney tissues, a total of 1025 miRNA editing sites were identified from 246 precursor miRNAs. Compared with normal kidney tissue samples, 122 editing events with significantly different editing levels were found in ccRCC, including two A-to-I editing events in the seed regions of has-mir-376a-3p and has-mir-376c-3p, and one C-to-U editing event detected in the seed region of has-mir-29c-3p, demonstrating the complexity and diversity of miRNA editing in ccRCC (23). The aforementioned related studies demonstrate the significant potential of RNA editing as a biomarker and therapeutic target in ccRCC.

This research will analyze the transcriptome and clinical data of ccRCC patients from the TCGA database. It will screen for prognosis-related RNA editing using unifactorial COX analysis, LASSO regression analysis, and multifactorial COX regression analysis. Finally, it will establish a training group and an internal validation group to evaluate the diagnostic and clinical utility of RNA editing in ccRCC patients using survival analysis and independent prognostic analysis, and validate it through *in vitro* experiments. Ultimately, the study will introduce a new concept for the development of gene therapy, targeted therapy, and individually tailored therapy for patients with ccRCC.

## 2 Materials and methods

### 2.1 Data collection

The TCGA database provided transcriptomic and clinical information for 614 samples, including 72 normal samples (normal human kidney tissue) and 542 KIRC samples (TCGA project ID:TCGA-KIRC).^1^ The Synapse database provided the TCGA-KIRC related RNA editing data (Project SynID: syn2374375)^2^ obtained as described in Han et al. (15). These samples were randomly assigned to two groups: a training group (*n* = 269) and a validation group (*n* = 179). The transcriptome data was arranged by Perl language, and the data analysis and mapping were realized by R package of R software.

### 2.2 Construct the prognostic model

Unifactorial COX analysis was used to screen the samples, and multifactorial COX analysis was then used for further screening. LASSO regression analysis was employed to identify associated genes. Seven RNA editing profiles (CHD3| chr17:7815229, MYO19| chr17.34853704, OIP5-AS1| chr15:41590962, MRI1| chr19:13883962, GBP4| chr1:89649327, APOL1| chr22:36662830, and FCF1| chr14:75203040) were found to be linked with prognosis. The genome version used hg19. The survival curves of the seven RNA editing profiles were shown in conjunction with the clinical survival data.

The pertinent risk coefficient values from multifactorial COX analysis results and the RNA editing expression levels were used to establish the risk score. The risk score for patients with ccRCC was determined using the following formula:

RiskScore = EXPgene(CHD3| chr17:7815229)*9.815022558 62103+EXPgene(MYO19| chr17:34853704)*6.79871066441166+ EXPgene(OIP5-AS1| chr15:41590962)*(-12.6723541150907)+EXP gene(MRI1| chr19:13883962)*2.37600410996414+EXPgene(GBP4| chr1:89649327)*(-4.43936413502299)+EXPgene(APOL1| chr22:36 662830)*5.71192867618665+EXPgene(FCF1| chr14:75203040)*6.8 0208663828929, where the numerical value is the risk coefficient and EXP represents the gene expression.

### 2.3 Evaluating prognostic models

Samples with incomplete clinical information (including TMN stage, age, gender, etc.) were deleted. Forest plots, unifactorial independent prognostic analysis, and multifactorial independent prognostic analysis were carried out based on the clinical information and risk scores of each sample. Analysis of the consistency index (C-index), calibration, time-dependent ROC curves, decision curves, and PFS survival curves were conducted. A nomogram created using the “Rms” program was used to predict the 1-, 2-, and 3-year survival rates of ccRCC patients.

For internal model validation, the 448 samples were divided into high and low-risk groups based on the median value of the risk score. These groups were then subjected to survival analysis, and risk and survival curves were plotted. Heat maps showing the expression of the RNA editing in the samples were applied to both the training group (*n* = 269) and the validation group (*n* = 179) to perform internal model validation.

### 2.4 Enrichment analysis

Genes in the high and low-risk groups underwent differential analysis. Volcano plots and heat maps were created for the top 50 differential genes, with filter conditions adjusted to | logFC| > 1 and FDR < 0.05 to obtain differential genes. GO and KEGG enrichment analysis was performed on the differential genes to examine variations in molecular processes and functional pathways. Box line plots were created to compare the expression differences of seven RNA editing between normal and tumor samples. Furthermore, the study compared risk scores and clinical features, used gene set enrichment analysis (GSEA) to analyze biological function differences, and analyzed the correlation between RNA editing and gene expression, as well as the correlation between risk scores and ADAR genes.

### 2.5 *In vitro* experimental validation

Human renal tubular epithelial cells HK-2 and ccRCC cells A-498, 786-O, and Caki-2 were provided by the ATCC and were grown in RPMI-1640 media with 10% FBS at 37°C in an incubator with 5% CO_2_. Total RNA was extracted from the transfected cells in each group using TRIzol reagent, and the Nano Drop 2000 system was used to measure the RNA’s concentration and purity. Whole RNA was reverse transcribed into cDNA using the Prime ScriptRT Master Mix reagent. The miScript SYBR Green PCR kit was utilized for quantitative polymerase chain reaction (qPCR), and the relative expression of the target genes was determined using the 2-ΔΔCt method, with β-actin serving as the internal reference gene. The primer sequences required for this investigation are listed in Table 1.

**TABLE 1:** Primer sequence.

RNA	Sequence (5′ to 3′)
β-actin	Forward Primer TCCGGCACTACCGAGTTATC Reverse Primer GATCCGGTGTAGCAGATCGC
CHD3| chr17:7815229	Forward Primer CCGTCAGCATTGGGTGTGAA Reverse Primer TCTTGCGTTTTCGGGGTTTTC
MYO19| chr17:34853704	Forward Primer GGGTGAATCCTGTGACACTAGA Reverse Primer GCCAGCATTGGTGTAGAATGT
OIP5-AS1| chr15:41590962	Forward Primer GTGTTGTGGAGATTGAGGCAGGAG Reverse Primer GGCAAGGTGAAGGACAGACAGC
MRI1| chr19:13883962	Forward Primer GATCCCCGCCACCCTTATC Reverse Primer GTCTCCAGACGGAGGTCACAT
GBP4| chr1:89649327	Forward Primer ATGGGTGAGAGAACTCTTCACG Reverse Primer TGCGGTATAGCCCTACAATGG
APOL1| chr22:36662830	Forward Primer TGGACTACGGAAAGAAGTGGT Reverse Primer CCTCCTTCAATTTGTCAAGGCTT
FCF1| chr14:75203040	Forward Primer AGGAAGTATGCGACCATGAAGC Reverse Primer AACGAGGATGTGGTAAGGTGG

### 2.6 Statistical analysis

The data were analyzed using the R program. Group differences were analyzed using one-way ANOVA. Two-by-two comparisons were conducted using the LSD test, unpaired *t*-test, and Wilcoxon test for non-normally distributed data. Differences were considered statistically significant when *P* < 0.05.

## 3 Results

### 3.1 Screening RNA editing profiles and constructing prognostic models

**FIGURE 1:**
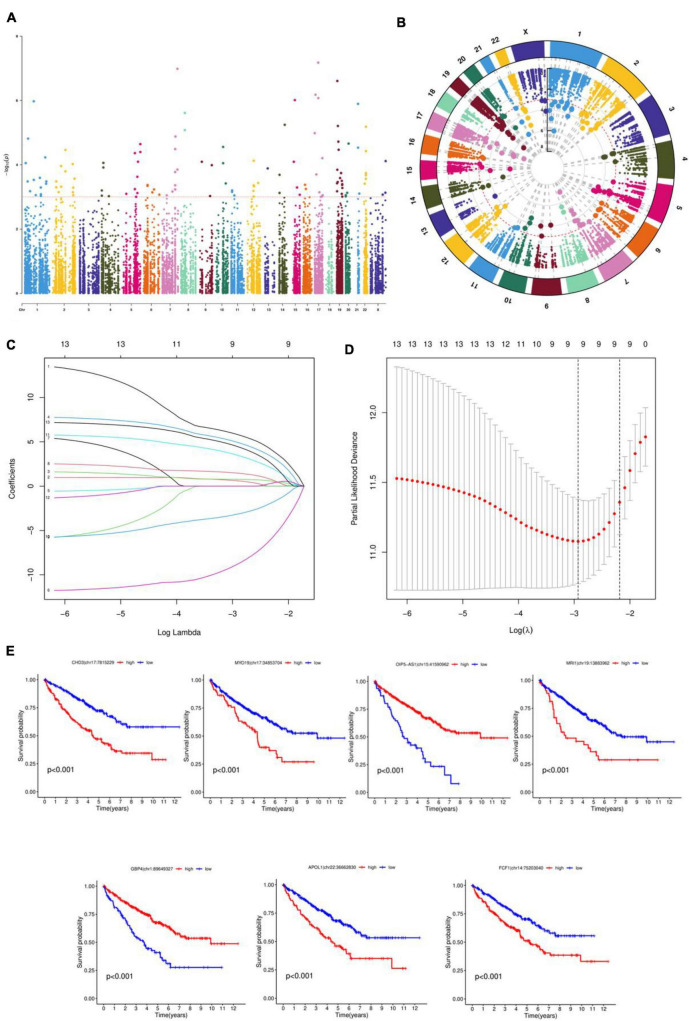
Screening RNA editing profiles and constructing prognostic models. **(A)** Manhattan diagram for RNA editing data. **(B)** Circle graph. **(C)** Ten-fold cross-validation for the coefficients in the LASSO model. **(D)** A coefficient profile plot was produced against the log (λ) sequence in the LASSO model. **(E)** Survival curves.

Figure 1A shows that samples with an RNA editing rate of less than 5% were excluded from the initial 63,717 data points of RNA editing sites collected, resulting in a total of 20,882 remaining data points. A unifactorial COX analysis was performed, and the results are presented in Figure 1B. The Manhattan plot was utilized to visualize the data with *P* < 0.001 significance. The data from the unifactorial COX analysis underwent LASSO regression, and the findings are illustrated in Figure 1C. To ensure the accuracy of the results and avoid overfitting, the regression parameters were adjusted 1,000 times for cross-validation. The LASSO model’s logarithmic (λ) sequences produced coefficient profiles, as shown in Figure 1D. Table 2 displays the results of the multifactorial COX regression analysis, highlighting seven significant RNA editing sites: MRI1| chr19:13883962, GBP4| chr1:89649327, APOL1| chr22:36662830, FCF1| chr14:75203040, CHD3| chr17:7815229, MYO19| chr17:34853704, OIP5-AS1| chr15:41590962. Plotting the survival curves in Figure 1E revealed that Patients with high expression of CHD3| chr17:7815229, MYO19| chr17:34853704, MRI1| chr19:13883962, APOL1| chr22:36662830 and FCF1| chr14:75203040 had higher survival rate, while patients with high expression of OIP5-AS1| chr15:41590962 and GBP4| chr1:89649327 had lower survival rates.

**TABLE 2:** Results of multifactorial COX regression analysis.

ID	Coef	HR	HR.95L	HR.95H	*P*-value
CHD3| chr17:7815229	9.815022559	31033556.91	34572.38131	27856966108	< 0.001
MYO19| chr17:34853704	6.798710664	7291.748144	289.1850817	183860.0756	< 0.001
OIP5-AS1| chr15:41590962	−12.67235412	0.000000013	0.000000001	0.000179865	< 0.001
MRI1| chr19:13883962	2.37600411	682.1990394	40.26762659	11557.56047	< 0.001
GBP4| chr1:89649327	−4.439364135	0.004552058	0.000522083	0.039689511	< 0.001
APOL1| chr22:36662830	5.711928676	676.8765148	39.79750187	11512.32602	< 0.001
FCF1| chr14:75203040	6.802086638	21095.89284	286.0605257	1555743.12	< 0.001

### 3.2 Evaluation of prognostic models

**FIGURE 2:**
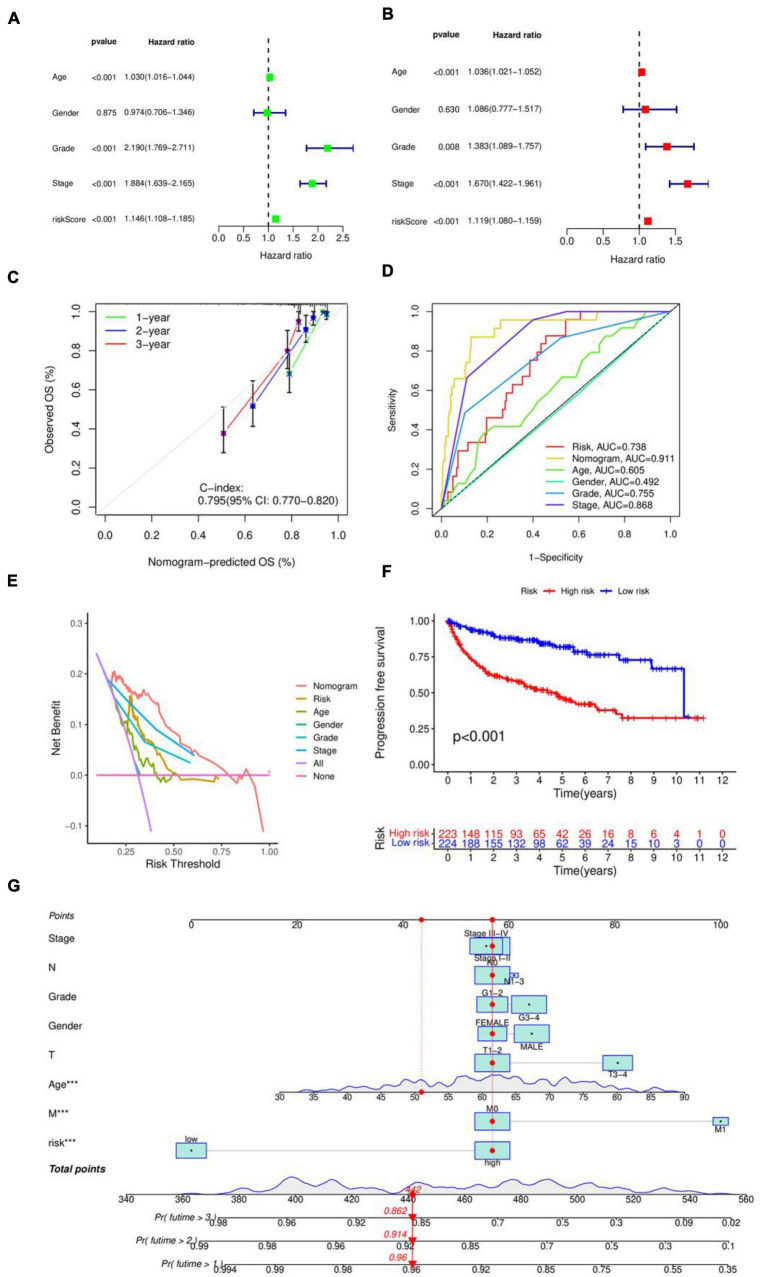
Evaluation of prognostic models. **(A)** Unifactorial independent prognostic analysis. **(B)** Multifactorial independent prognostic analysis. **(C)** Consistency index analysis. **(D)** ROC curves. **(E)** Decision curves. **(F)** PFS survival curves. **(G)** Nomogram for predicting patients survival rate.

The risk score in the prognostic model primarily conveys the probability or degree of risk of an individual experiencing a certain adverse outcome (such as disease recurrence, death, disability, or complications) within a future period. This score is derived from a multifactorial model that typically considers various factors influencing the individual’s prognosis, including disease type, disease stage, patient age, gender, treatment modalities, and so forth. The results in Figures 2A, B of the univariate and multivariate independent prognostic analyses indicate that age, stage, grade, and especially the risk score, can serve as independent prognostic factors. Figure 2G creates a nomogram combining risk score with clinical characteristics, with predictive accuracies of 0.96, 0.914, and 0.862 for 1, 2, and 3 years, respectively. The C-index value of the concordance curve in Figure 2C is greater than 0.7, the AUC value of the ROC curve in Figure 2D is 0.738, and the decision curve in Figure 2E confirms the accuracy of the nomogram. Figure 2F shows the results of an PFS survival curve, suggesting that patients at higher risk had reduced survival rates.

### 3.3 Validation of internal models

**FIGURE 3:**
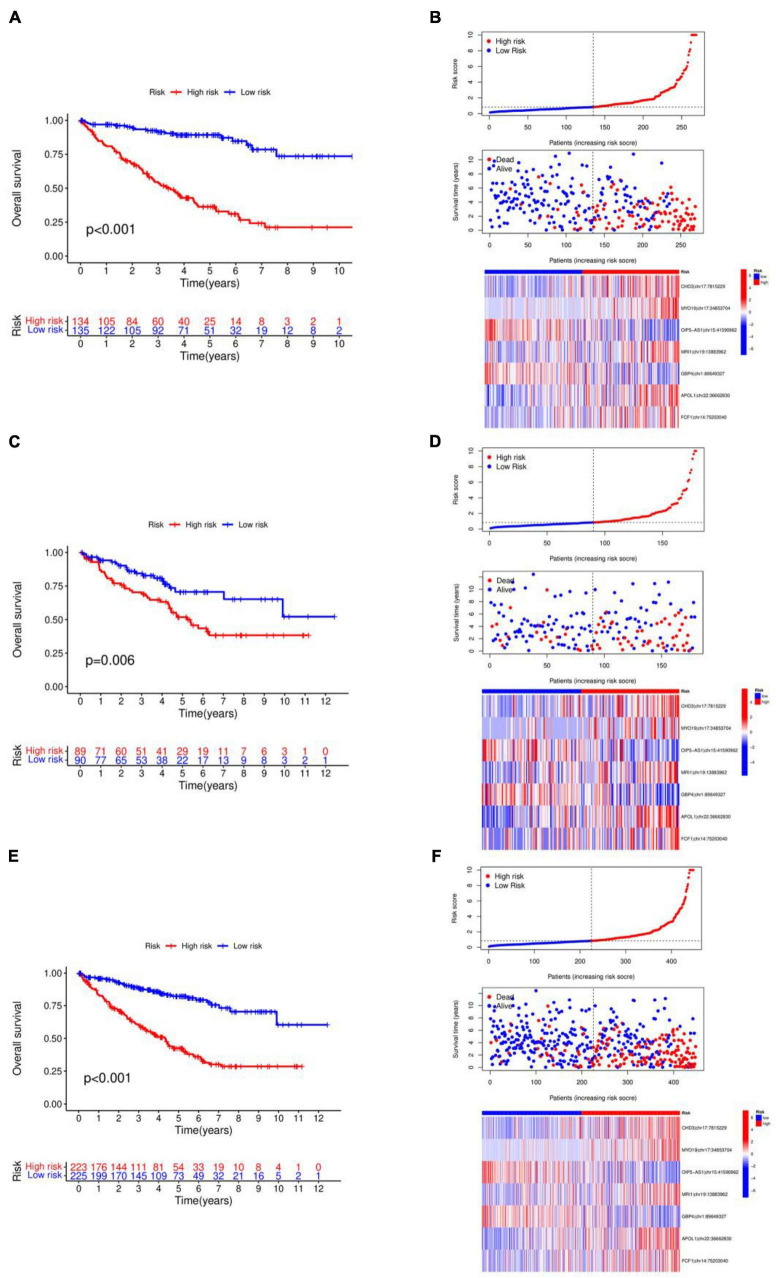
Validation of internal models. **(A)** Kaplan–Meier curve in the training group. **(B)** Survival state chart, risk curve and heatmap in the training group. **(C)** Kaplan–Meier curve in the validation group. **(D)** Survival state chart, risk curve and heatmap in the validation group. **(E)** Kaplan–Meier curve in the combination set. **(F)** Survival state chart, risk curve and heatmap in the combination set. The number of patients who passed away grew as the risk score increased.

The 448 samples were divided into high and low-risk groups based on the median risk score. This allowed for the construction of the internal model validation in both the training group (*n* = 269) and the validation group (*n* = 179), the drawing of risk curves, and the creation of a heat map. Figures 3A, C, E display the survival curve, showing that patients in the high-risk group had a worse prognosis for survival, indicating that the model accurately predicts survival and prognosis, in line with the prognostic model’s forecast. Figures 3B, D, F display the risk curves, indicating that the number of patients who passed away grew as the risk score increased. The heat map results identified OIP5-AS1| chr15:41590962 and GBP4| chr1:89649327 as protective factors, and CHD3| chr17:7815229, MYO19| chr17:34853704, MRI1| chr19:13883962, APOL1| chr22:36662830, and FCF1| chr14:75203040 as risk factors.

### 3.4 Variance and enrichment analysis

**FIGURE 4:**
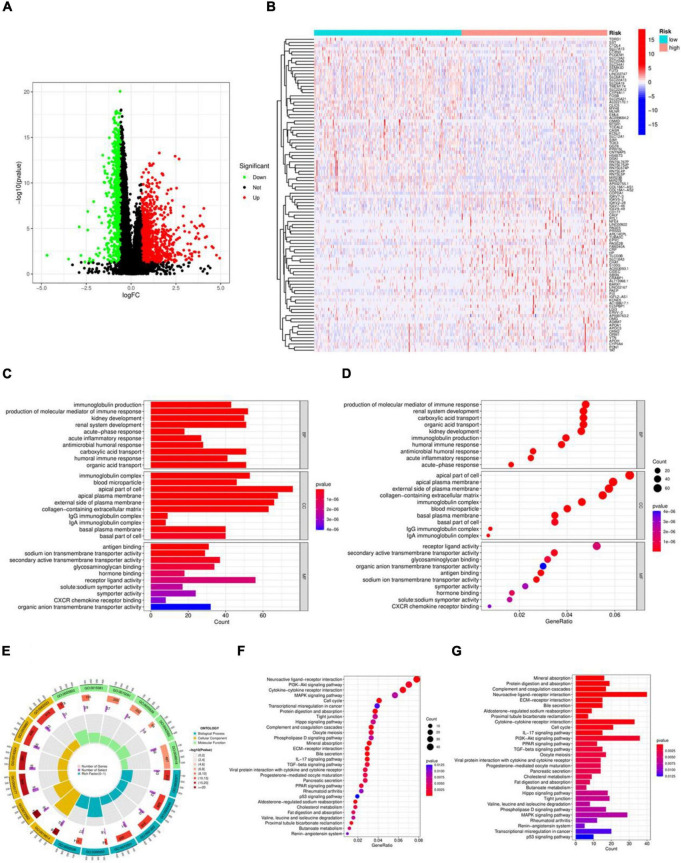
Variance and enrichment analysis. **(A)** Volcano plot. **(B)** Heatmap of the top 50 differentially expressed genes. **(C)** Histogram of the GO enrichment analysis. **(D)** Bubble plot of the GO enrichment analysis. **(E)** Circle plot of the GO enrichment analysis. **(F)** Bubble plot of the KEGG enrichment analysis. **(G)** Histogram of the KEGG enrichment analysis.

The differential genes of the high and low risk groups were analyzed, as shown in the volcano map in Figure 4A, showing 230 differentially expressed genes. According to *P* < 0.05, | log_2_FC| > 1.5, 208 genes were significantly up-regulated and 22 genes were down-regulated in the high risk group. Figure 4B shows a heatmap of the top 50 differentially expressed genes. The results of GO enrichment analysis in Figures 4C–E indicate that, in terms of Biological Process (BP), the differentially expressed genes were mainly enriched in immunoglobulin production, production of molecular mediators of immune response, and kidney development. Regarding Cellular Component (CC), the differentially expressed genes were mainly enriched in immunoglobulin complex, blood microparticle, and apical part of cell. In the case of Molecular Function (MF), the differentially expressed genes were mainly enriched in antigen binding, sodium ion transmembrane transporter activity, and secondary active transmembrane transporter activity. The KEGG enrichment analysis results in Figures 4F, G show that the differentially expressed genes were mainly enriched in Neuroactive ligand-receptor interaction, PI3K-Akt signaling pathway, and Cytokine-cytokine receptor interaction.

### 3.5 Clinical characterization analysis

**FIGURE 5:**
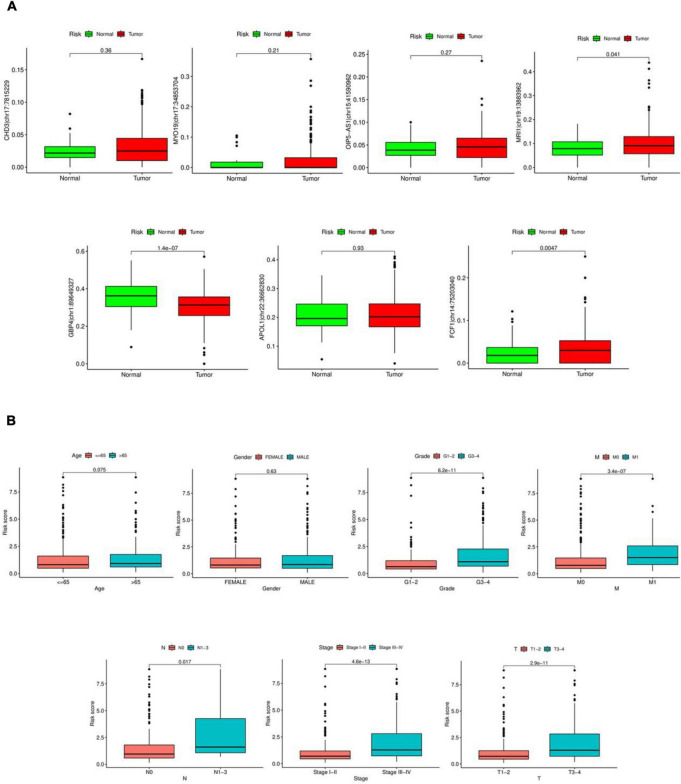
Clinical characterization analysis. **(A)** Expression of seven RNA editing sites in tumor and normal samples. Red is tumor sample, green is normal sample. **(B)** Differential analysis of risk scores in clinical characteristics.

As shown in Figure 5A, significant differences were observed between tumor and normal samples for MRI1| chr19:13883962, GBP4| chr1:89649327, and FCF1| chr14:75203040. However, no significant differences were found between tumor and normal samples for OIP5-AS1| chr15:41590962, CHD3| chr17:7815229, MYO19| chr17:34853704, and APOL1| chr22:36662830. Risk scores showed considerable variation in Grade, M-stage, N-stage, T-stage, and Stage, but did not differ significantly by Age or Gender, as illustrated in Figure 5B.

### 3.6 GSEA enrichment analysis and correlation analysis

**FIGURE 6:**
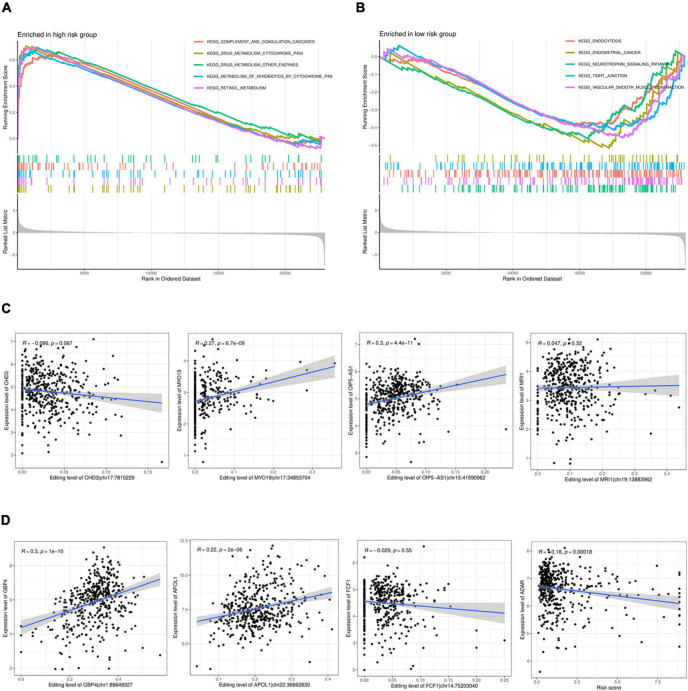
GSEAenrichment analysis and correlation analysis. **(A,B)** GSEAenrichment analysis. **(C)**. Analysis of the correlation between the level of RNA editing and the corresponding gene expression. **(D)** Analysis of the correlation between the expression in ADAR and the risk score.

The GESA enrichment results in Figure 6A demonstrate that complement and coagulation cascades, drug metabolism cytochrome P450, drug metabolism other enzymes, metabolism of xenobiotics by cytochrome P450, and retinol metabolism were significantly active in the high-risk group. In contrast, the results in Figure 6B indicate that endocytosis, endometrial cancer, neurotrophin signaling pathway, tight junction, and vascular smooth muscle contraction were significantly active in the low-risk group. Given that ADAR acts as the primary mediator of RNA editing, the results in Figure 6D show a substantial connection (*r* = 0.18, *P* < 0.001) between the risk score and ADAR gene expression. The findings presented in Figure 6C demonstrate a significant correlation between the degree of RNA editing of MYO19| chr17:34853704, OIP5-AS1| chr15:41590962, GBP4| chr1:89649327, APOL1| chr22:36662830, and the expression of their corresponding genes.

### 3.7 qPCR results

**FIGURE 7:**
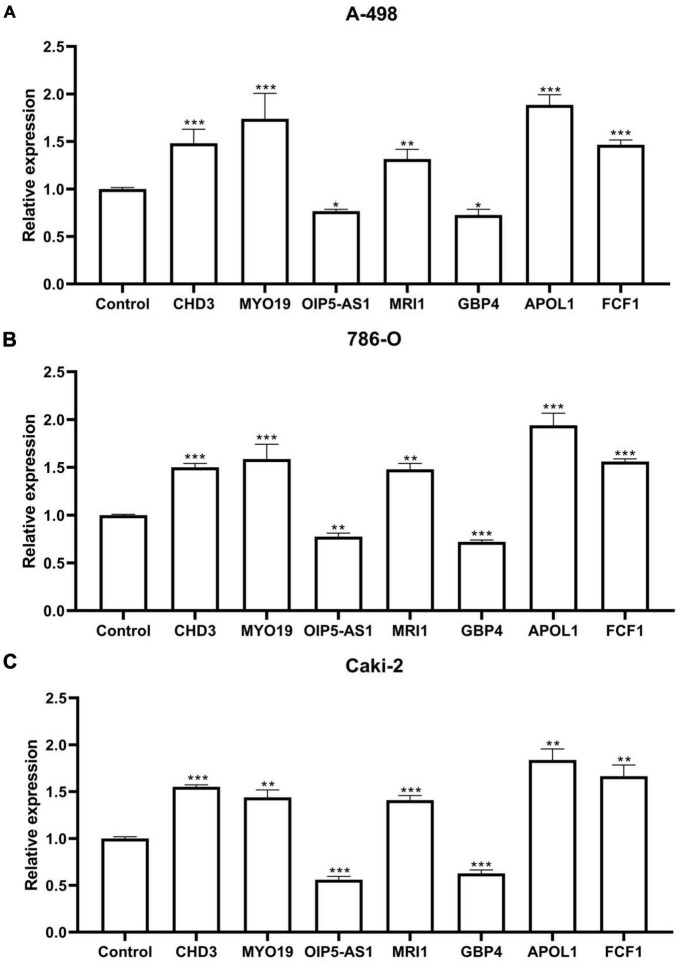
qPCR results. **(A)** Seven RNA editing sites expressed in the ccRCC cell line A-498. **(B)** Seven RNA editing sites expressed in the ccRCC cell line 786-O. **(C)** Seven RNA editing sites expressed in the ccRCC cell line Caki-2. Compared with control group, *P < 0.05; **P < 0.01; ***P < 0.001.

The Figures depicted in Figures 7A–C demonstrate that the mRNA expression levels of CHD3, MYO19, MRI1, APOL1, and FCF1 were significantly up-regulated in the ccRCC cell lines A-498, 786-O, and Caki-2 compared to the control group. Conversely, the mRNA expression levels of OIP5-AS1 and GBP4 were significantly down-regulated. These differences were statistically significant, Each cell line was controlled by normal renal tubular epithelial cells HK-2, which measured β-actin mRNA expression.

## 4 Discussion

Many individuals are not diagnosed with kidney cancer until it has progressed to an advanced stage due to the subtle and generic clinical symptoms and indicators of the disease in its early stages (24). The most significant pathological subtype of renal cancer is ccRCC. This prevalent form of renal cell carcinoma lesions begins in the proximal tubules and is characterized by a thin-walled vascular network and a high proportion of clear cells (25). Chemotherapy and radiation treatment have little effect on ccRCC, and its rate of recurrence and metastasis is significantly higher. Patients with early-stage ccRCC have a 5-year overall survival rate of up to 90%, while patients with locally progressed and metastatic ccRCC have 5-year overall survival rates of 50 and 10%, respectively (26, 27). For individuals with ccRCC, a precise prognosis and prompt diagnosis are crucial.

Bioinformatics is a cross-disciplinary field that encompasses the collection, management, preservation, distribution, analysis, and understanding of biological data to uncover and interpret the biological meanings of a vast amount of scientific data (28). It also integrates the use of mathematical, computer, and biological tools. Bioinformatics has the ability to extract useful information for humans from the abundance of biological data, enabling them to more effectively address pertinent biological issues (29). Fundamental research on the actions of cancer factors has been vigorously conducted in the field of bioinformatics, particularly in genomics and proteomics, due to the progress of sequencing technology and the human genome project in several nations (30). Researcher participation in the analysis of biological data, the development of untested and unexamined information in biological data, and the execution of follow-up external experimental studies have all been made possible by the growing quantity and quality of public databases, thus contributing to the advancement of various disciplines and fields of research (31, 32).

Due to advancements in next-generation sequencing technology, over a million A-to-I RNA editing sites have been detected. A significant portion of these editing sites reside in non-coding and repetitive element regions, and their functions remain largely unknown (33). However, research suggests that A-to-I RNA editing plays a crucial role in cancer prognosis and predicting survival (34). Researchers have obtained glioma genome and clinical data from the TCGA database and Synapse platform. By employing regression analysis, they have identified RNA editing sites related to prognosis and calculated their corresponding risk coefficients. The results indicate that a higher risk score correlates with poorer prognosis, weaker immune response, and lower sensitivity to immunotherapy. The characteristics of prognosis-related RNA editing sites can aid in risk stratification, prediction of immunotherapy response, development of personalized treatment strategies for glioma patients, and discovery of novel therapeutic approaches (35). To investigate the impact of A-to-I RNA editing on the prognosis of bladder cancer patients, researchers obtained gene expression and clinical data from 251 patients in the TCGA database. They randomly divided the patients into training and testing groups. By identifying A-to-I RNA editing sites associated with prognosis, they constructed a prognostic model and generated risk scores. Patients with higher scores exhibited significantly worse OS compared to those with lower scores. Additionally, nomograms combined with the scores provided improved prediction of patient prognosis. Various functional and pathway changes related to immune response, as well as significant differences in immune cell infiltration levels and drug treatment response, were observed between high- and low-scoring patients (36). In a related study on A-to-I RNA editing sites associated with survival in lung adenocarcinoma, 10441 A-to-I RNA editing site data from 440 LUAD patients in the TCGA database were evaluated. The ATIRE landscape was merged with TCGA survival data. Tumor staging and risk scores in lung adenocarcinoma patients were associated with OS. Notably, the level of A-to-I RNA editing in tumor tissues was significantly elevated, showing considerable variability among patients. This suggests that A-to-I RNA editing can serve as a unique predictor of lung adenocarcinoma survival rates (37).

The RNA editing profiles associated with the prognosis of ccRCC were obtained through unifactorial cox analysis, multifactorial cox analysis, and lasso regression analysis. These RNA editing profiles were CHD3| chr17:7815229, MYO19| chr17.34853704, OIP5-AS1| chr15:41590962, MRI1| chr19:13883962, GBP4| chr1:89649327, APOL1| chr22:36662830, FCF1| chr14:75203040. The survival curves indicated that the expression of the aforementioned seven RNA editing sites was significantly different in ccRCC patients. After calculating risk scores, prognostic models were built. The findings of unifactorial and multifactorial independent prognostic analyses indicated that risk scores could be used as independent prognostic factors. We also constructed PFS survival curves, which showed that patients at higher risk had a lower survival rate. Nomograms were used to predict patient survival, and their accuracy was confirmed by concordance, ROC, and decision curves.

This research constructed an internal validation model, applied risk scores to the training and validation groups, plotted survival curves and risk curves, and created heat maps of the expression of RNA editing sites in the samples. The results aligned with the risk model, with patients in the high-risk group having a worse prognosis for survival, indicating that the model can predict survival prognosis more accurately. Additionally, as the risk scores increased, the number of deaths increased, aligning with the model’s forecast. The results of the risk heat map showed that OIP5-AS1| chr15:41590962 and GBP4| chr1:89649327 were protective factors, while CHD3| chr17:7815229, MYO19| chr17:34853704, MRI1| chr19:13883962, APOL1| chr22:36662830, and FCF1| chr14:75203040 were risk factors.

OIP5-AS1, a conserved lncRNA located on chromosome 15q15.1, is involved in a variety of biological and pathological processes, several studies have suggested that OIP5-AS1 may act as an oncogene for specific cancer types (38). Researcher found that OIP5-AS1 expression was down-regulated in tissues affected by multiple myeloma (MM), and in MM cells, overexpression of OIP5-AS1 demonstrated anti-tumor potential (39). The GTPase class including guanylate-binding proteins (GBP) is essential for both host cell immunity and antimicrobial defense. They detect infections and stop germs from growing by controlling cellular pyroptosis and triggering inflammatory vesicles (40). GBP4 is implicated in pathological processes such as tumorigenesis and progression. Prognostic prediction models constructed with GBP4 were used to evaluate the prognosis of melanoma patients. High expression of GPB4 has been associated with excellent overall survival of more than 30 years in individuals with cutaneous melanoma (41). The CHD3 gene is located at 17p13.1 and has 40 exons and 7356 bases. The CHD3 protein, a member of the chromatin domain deconjugating enzyme DNA-binding protein family, contains one ATP-binding deconjugate enzyme region, two finger plant homology domains, and two chromatin domains (42). One study used an integrated bioinformatics method to identify CHD3 as a hub gene associated with the pathophysiology of Alzheimer’s disease (43). MYO19 is a member of the class 19 subgroup of the myosin superfamily, sharing a conserved, plus end-directed motor structural domain, a lever arm containing three light chain-binding IQ motifs, and a unique tail region known as the Myosin Mitochondrial Outer Membrane-Associated (MOMA) structural domain. This domain directs MYO19 to mitochondria and plays a key role in mitochondrial partitioning, regulation of fission and fusion homeostasis (44, 45). Studies have shown that dysregulation of MYO19 is associated with gliomas and breast cancer (46, 47). One key amino acid in the methionine recycling process is methionine, catalyzed by the methyl thioredoxin-1-phosphate isomerase 1 (MRI1). Methionine is essential for the growth of several malignancies, including gliomas, bladder cancer, breast cancer, melanoma, and prostate cancer (48). Research has demonstrated that an increase in the methionine metabolic pathway is linked to the metastasis and development of ccRCC (49). The APOL1 gene product may lead to mitochondrial dysfunction through numerous pathways. The five-domain protein APOL1 has various intracellular roles, and cellular stressors such as inflammatory signals, food restriction, and hypoxia increase its production. The pH-dependent colistin-like pore-forming structural domain of APOL1 can be incorporated into lysosomes, cell membranes, or the mitochondrial phospholipid bilayer. Pore formation is enabled by G1 and G2 mutations at reduced APOL1 gene expression levels. Due to lysosomal and autophagic flux abnormalities, APOL1 may cause direct or indirect damage to mitochondria (50). It has been demonstrated that APOL1 may function as an oncogene to stimulate proliferation and block apoptosis by triggering the expression of the NOTCH1 signaling pathway in pancreatic cancer. As a result, it may offer a potential therapeutic target for the disease (51). Although less research has been done on FCF1, a ribosome biogenesis factor with a PIN nucleic acid endonuclease structural domain, it is particularly significant for RNA cleavage of eukaryotic early pro-ribosomes (52).

The volcano plot showed the inclusion of 208 up-regulated genes and 22 down-regulated genes. A GO enrichment analysis was conducted to investigate the molecular processes and signaling pathways of the differential genes. The results indicated that, in terms of BP, the differential genes were primarily enriched in immunoglobulin production, production of molecular mediators of immune response, and kidney development. In terms of CC, the differential genes were mainly enriched in immunoglobulin complex, blood microparticle, and apical part of the cell. In terms of MF, the differential genes were mainly enriched in antigen binding, sodium ion transmembrane transporter activity, and secondary active transmembrane transporter activity. KEGG enrichment analysis revealed that the differential genes were mainly enriched in Neuroactive ligand-receptor interaction, PI3K-Akt signaling pathway, and Cytokine-cytokine receptor interaction. GSEA enrichment analysis showed significant activity of complement and coagulation cascades, drug metabolism cytochrome P450, and other pathways in the high-risk group, while pathways such as endocytosis and neurotrophin signaling were significantly active in the low-risk group.

ADAR-mediated RNA editing is crucial for mammalian survival, and dysregulation can lead to the formation of lesions (53). Studies have shown that ADAR-induced substitution of Ser367gly at the locus of antitumor enzyme inhibitor 1 (AZIN1) increases the binding affinity of AZIN1 and inhibits its ability to inhibit ornithine decarboxylase, resulting in the development of more tumorigenic characteristics in hepatocellular carcinoma (54). There is a significant association between risk ratings and ADAR gene expression, as found using correlation analysis. Additionally, correlation analysis revealed a strong link between the corresponding genes and the degree of RNA editing of MYO19| chr17:34853704, OIP5-AS1| chr15:41590962, GBP4| chr1:89649327, and APOL1| chr22:36662830.

Finally, using ccRCC cell lines A-498, 786-O, and Caki-2, qPCR results from *in vitro* experiments showed that the mRNA expression levels of CHD3, MYO19, MRI1, APOL1, and FCF1 were significantly up-regulated compared to the control group. Additionally, the mRNA expression levels of OIP5-AS1 and GBP4 were significantly down-regulated compared to the control group.

## 5 Conclusion

To sum up, CHD3| chr17:7815229, MYO19| chr17:34853704, OIP5-AS1| chr15:41590962, MRI1| chr19:13883962, GBP4| chr1:89649327, APOL1| chr22:36662830, FCF1| chr14:75203040 represent seven RNA editing sites that were screened. These sites are expected to serve as potential biomarkers for ccRCC. This research will provide a new approach for personalized treatment and prognosis evaluation for ccRCC patients.

## Data availability statement

The original contributions presented in this study are included in this article/supplementary material, further inquiries can be directed to the corresponding author.

## Ethics statement

Ethical approval was not required for the study involving humans in accordance with the local legislation and institutional requirements. Written informed consent to participate in this study was not required from the participants or the participants’ legal guardians/next of kin in accordance with the national legislation and the institutional requirements. The manuscript presents research on animals that do not require ethical approval for their study.

## Author contributions

WC: Conceptualization, Data curation, Formal analysis, Investigation, Methodology, Project administration, Software, Supervision, Validation, Writing – original draft, Writing – review & editing. SL: Conceptualization, Data curation, Formal analysis, Investigation, Methodology, Project administration, Software, Supervision, Validation, Writing – original draft, Writing – review & editing. DH: Formal analysis, Funding acquisition, Methodology, Project administration, Resources, Supervision, Validation, Visualization, Writing – original draft, Writing – review & editing. YS: Conceptualization, Data curation, Investigation, Methodology, Software, Supervision, Writing – original draft, Writing – review & editing. JW: Conceptualization, Data curation, Formal analysis, Investigation, Methodology, Project administration, Software, Supervision, Validation, Writing – original draft. ZL: Conceptualization, Data curation, Formal analysis, Funding acquisition, Investigation, Methodology, Project administration, Resources, Software, Supervision, Validation, Visualization, Writing – original draft, Writing – review & editing.

## Footnotes

1
https://portal.gdc.cancer.gov/

2
https://www.synapse.org/#!Synapse:syn2374375/files/
